# Controls and Result Interpretations in Studies of Urine Gluten Peptide Determinations

**DOI:** 10.14309/ctg.0000000000000456

**Published:** 2022-01-19

**Authors:** Carolina Sousa, Isabel Comino, Ángel Cebolla, Laura Coto, Verónica Segura, Ángela Ruiz-Carnicer, María de Lourdes Moreno

**Affiliations:** 1 Department of Microbiology and Parasitology, Faculty of Pharmacy, University of Seville, Seville, Spain;; 2 Biomedal S.L., Seville, Spain.

We have read with interest the article by Monachesi et al. (1), in which the authors assess the diagnostic performance of urinary gluten immunogenic peptides (GIP) determination to detect gluten contamination of the gluten-free diet (GFD) in a group of healthy and qualified Italian volunteers adhering to GFD and undergoing repeated dietary challenges with increasing amounts of gluten. In contrast to over a dozen studies from multiple groups reporting the validity, utility, and reliability of the GIP tests in the monitoring of the GFD (2), this is the first study to date suggesting that the urine GIP test was not reliable because of a high frequency of reported false positive and negative determinations. Although it was not mentioned how they measured the GIP with the lateral flow immunoassays, they did not find the expected correlation between the ingested gluten (10, 50, 100, 500, and 1,000 mg) and the amount of excreted GIP, with significant negative results for the 1,000-mg ingestion.

Considerable rates of GIP positivity similar to these claimed as “false positives” here have been previously reported in celiac patients on a GFD (3,4). Other studies with trained volunteers undergoing GFD showed absence of positive excreted GIP or few positives from identified accidental ingested gluten (3,4). It is entirely feasible that the positives interpreted as “false positives” in study A were due to gluten contamination or transgressions in the GFD, as confirmed with the strictest study B of the same authors. They did not take any standard measure to back their interpretation of the positive determinations as “false positives,” for example, collecting the foodstuffs consumed by the study subjects and quantifying their gluten content, as described by Sylvester et al. (4). They could have supplied the food daily or used the more sensitive stool GIP tests to control the GFD of the volunteers (5). Qualitative testing with the urine tests should have been considered to support the quantitative data supposedly made with a lateral flow reader, which requires calibration per batch. After having inconsistent quantitative results, the capacity to measure urine GIP should have been confirmed using calibrators and spiked samples in negative urine.

In study B, it is normal to find negative GIP tests in urine when the volunteers ingested 5 or 10 mg of gluten. However, it was not clear why they used the urine tests to detect levels of GIP below the manufacturer specifications which are indicated for detection of 50–500 mg gluten consumption.

In conclusion, the suggestions of Monachesi et al. (1) of false positive and negative results in their study were likely misinterpreted, and we encourage the authors to conduct the appropriate controls.

## CONFLICTS OF INTEREST

**Guarantor of the article:** Carolina Sousa, PhD.

**Specific author contributions:** C.S., I.C., Á.C., L.C., V.S., A.R.-C. and M.L.M.: data collection and interpretation; manuscript preparation and review.

**Financial support:** None to report.

**Potential competing interests:** A.C. is the founder and current CEO of Biomedal S.L. L.C. is an employee and PhD student at Biomedal S.L. The method of this letter was included in a patent application (No. P201400569) by C.S., A.C., and M.L.M. as inventors. Other authors have declared no conflict of interest.
